# The effect of pre-existing sarcopenia on outcomes of critically ill patients treated for COVID-19

**DOI:** 10.2478/jccm-2024-0045

**Published:** 2025-01-31

**Authors:** Thomas Bradier, Sébastien Grigioni, Céline Savoye-Collet, Gaétan Béduneau, Dorothée Carpentier, Christophe Girault, Maximillien Grall, Grégoire Jolly, Najate Achamrah, Fabienne Tamion, Zoé Demailly

**Affiliations:** Intensive Care Unit, Charles Nicolle University Hospital, Rouen, France; Department of Nutrition, Charles Nicolle University Hospital, Rouen, France; Radiology Department, QUANTIF-LITIS EA 4108, Charles Nicolle University Hospital, Rouen, France; Normandie Univ, UNIROUEN, UR 3830-GRHVN, CHU Rouen, F-76000 Rouen, France; Normandie Univ, UNIROUEN, INSERM U1096, CHU Rouen, France

**Keywords:** COVID-19, Skeletal Muscle Index, sarcopenia, sarcopenic obesity, death, Intensive Care Unit

## Abstract

**Background:**

Sarcopenia, defined by a loss of skeletal muscle mass and function, has been identified as a prevalent condition associated with poor clinical outcome among critically ill patients. This study aims to evaluate the impact of pre-existing sarcopenia on outcomes in critically ill patients with acute respiratory failure (ARF) due to COVID-19.

**Material and Methods:**

A retrospective study was carried out on COVID-19 patients admitted to intensive care. Pre-existing sarcopenia was assessed using early CT scans. Clinical outcomes, including duration of high-flow oxygenation (HFO), mechanical ventilation (MV), length of hospital stay (LOS) and ICU mortality, were evaluated according to sarcopenia status.

**Results:**

Among the studied population, we found a high prevalence (75 patients, 50%) of pre-existing sarcopenia, predominantly in older male patients. Pre-existing sarcopenia significantly impacted HFO duration (6.8 (+/−4.4) vs. 5 (+/−2.9) days; p=0.005) but did not significantly affect MV requirement (21 (28%) vs. 23 (37.3%); p=185), MV duration (7 vs. 10 days; p=0.233), ICU mortality (12 (16%) vs. 10 (13.3 %); p=0.644) or hospital LOS (27 vs. 25 days; p=0.509). No differences in outcomes were observed between sarcopenic and non-sarcopenic obese patients.

**Conclusions:**

Pre-existing sarcopenia in critically ill COVID-19 patients is associated with longer HFO duration but not with other adverse outcomes. Further research is needed to elucidate the mechanisms and broader impact of sarcopenia on septic critically ill patient outcomes.

## Background

Sarcopenia was defined by the European Working Group on Sarcopenia in Older People in 2019 as an impairment of muscle quality combined with a loss of muscle strength [1]. Aging, illness, physical inactivity, and malnutrition are strongly associated with its occurrence [2]. In critical care patients, these elements are frequently encountered, and sometimes even combined. Observational studies have reported a high prevalence of sarcopenia in hospitalized patients, up to 60% of those admitted to intensive care unit (ICU) [3], which appears to be associated to increased risks of prolonged hospital length of stay (LOS), readmission and mortality in this population [4,5]. This severe condition can be objectively defined by measuring muscle mass on computed tomography (CT) scans [6], and is strongly associated with mortality in patients with sepsis [7]. Sarcopenia has been widely described in patients with COVID-19, with muscle loss of up to 30% in 10 days in patients with severe forms [8]. Another study reported a loss of muscle function of up to 80%, with associated muscle mass loss, in patients without previous disabilities [9]. Indeed, during SARS-CoV-2 infection, two major factors are linked to the development of sarcopenia : undernutrition [10], and inflammation, described as an uncontrolled and excessive release of cytokines, known as a “cytokine storm” [11]. In sepsis, inflammatory cytokines activate numerous signaling pathways involved in muscle protein degradation, overriding protein synthesis and leading to muscle wasting [12]. Although there seems to be a strong relationship between sarcopenia and COVID-19 severity, these studies on muscle mass or malnutrition in COVID-19 did not report temporal changes in muscle mass and their impact on clinical outcomes [13]. The impact of pre-existing sarcopenia on the prognosis of patients admitted to ICU remains to be determined to consider nutritional strategies in the future.

The aim of this study was to assess the prevalence of pre-existing sarcopenia on ICU admission and its impact on outcomes in critically ill patients admitted to ICU for hypoxemic acute respiratory failure (ARF) due to COVID-19.

## Methods

### Study design and setting

This is a retrospective cohort study conducted in a French tertiary care center. The study was approved by the internal review board (N° 2022/0081/OB) and by the Commission for the Qualification of Research Projects of Rouen University Hospital (N°595). It was conducted in accordance with the Declaration of Helsinki.

### Study Population

The study included consecutive patients admitted to ICU for hypoxemic ARF with reverse transcription polymerase chain reaction (RT-PCR) confirmed COVID-19, and available abdominal or thoracic CT scan performed within seven days of ICU admission.

Patients without biological confirmation of COVID diagnosis, pregnant women, and patients whose CT scan could not be interpreted according to sarcopenia criteria were excluded.

### Data collection

All data were collected from patients' electronic health records. The following data were collected at baseline: age, sex, weight, Body Mass Index (BMI), comorbidi-ties, Sequential Organ Failure Assessment (SOFA) score, Simplified Acute Physiology Score (SAPS II), biological parameters including lymphocyte, albumin, C reactive protein (CRP), D-dimer, and fibrinogen levels. ICU mortality, occurrence of pneumonia, use of high flow oxygenation (HFO) and mechanical ventilation (MV), lung damage, defined as mild, moderate, severe or very severe depending on the surface area affected (<25%, 25–50%, 50–75% or >75% respectively), ICU and hospital LOS were also recorded.

### Body composition measurements

CT scan is considered as one of the most accurate methods available for conducting body composition analysis [14]. As reported in other studies, muscle mass and fat tissue were measured by analyzing stored images from available imaging performed before or during the first 3 days of ICU admission [15]. On a single CT image, skeletal muscle and fat tissue area were segmented by a dedicated plugin running on Carestream - Picture Archiving and Communication System. Hounsfield unit (HU) - based image analysis was performed using dedicated macro-Image software [16], to segment fat and lean tissue and quantify the cross-sectional area (CSA) (cm^2^) of each tissue type by summing the pixels of a given tissue and multiplying the sum by the absolute unit pixel surface area [17,18]. CT CSA of tissue was identified through HU measured from −29 to 150 for skeletal muscle and −190 to −30 and −50 to −150 for subcutaneous and visceral adipose tissue, respectively [19].

The quality criteria of the CT sections [19] had to be validated by two readers (TB and CSC); otherwise, the images were excluded from the analysis. Each tissue was examined by the two readers (TB and CSC). After quality control, the index values of one reader (TB) were used for further statistical tests.

For the study, we assessed the skeletal muscle and subcutaneous fat areas at the level of the twelfth thoracic vertebra (T12), in axial section using thoracic CT. The T12 skeletal muscles included erector spinae, latissimus dorsi, rectus abdominis, obliquus externus, internus abdominis, and internal and external intercostal muscles.

### Definition of sarcopenia

The Skeletal Muscle Index (SMI) is used to assess muscle mass and determine sarcopenia during a standard CT scan. This method was initially used for cancer patients [1] and developed for measurements at the L3 level. It has recently been validated in T12 [6]. The SMI corresponds to the Skeletal Muscle Area (SMA, i.e. muscular surface measured by CT scan) indexed to the size squared, allowing a reflection of the muscular composition in relation to body surface area. At the level of T12, we employed cut-off values of SMA <56.1 cm^2^ and SMI<20.8 cm^2^/m^2^ in women, and SMA <92.3 cm^2^ and SMI<28.9 cm^2^/m^2^ in men [6].

### Study outcomes

The primary outcome was the prevalence of pre-existing sarcopenia on ICU admission in critically ill patients admitted for hypoxemic ARF due to COVID-19.

Secondary outcomes were ICU and hospital mortalities, acquired pneumonia within 48 hours, defined as purulent sputum or tracheal aspiration, declining oxygenation or increased oxygen requirement, and new or progressive lung infiltrates on chest radiographs; the use and duration of HFO and MV; and the ICU and hospital LOS.

### Statistical analysis

Data are presented as mean values (+/− SD) for continuous variables, and categorical data are represented as frequencies and percentages. Chi-squared tests were used for categorical variables and t-test or ANOVA for continuous variables. Tests were two-sided, and the significance threshold was set at 0.05. All statistical analysis was performed with SPSS software version 20.0.

## Results

**Fig. 1.**
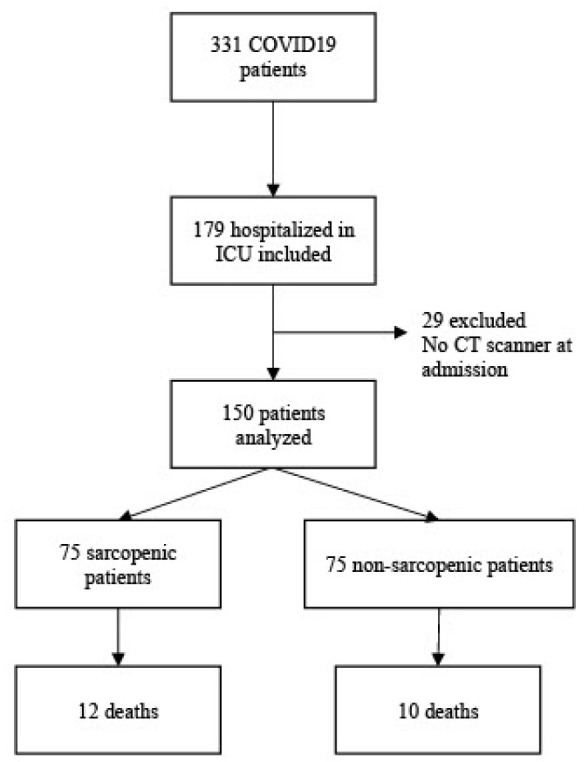
Flow chart

Between March 1^st^ 2020 and September 30th 2020, 331 patients were admitted to our center de novo COVID-19 infection. Among them, 179 patients were admitted to the ICU. Finally, 150 ICU patients with ARF secondary to COVID-19 and an initial CT scan were included in the analysis (Figure 1).

### Patients' characteristics at baseline (Table 1)

The population was mainly composed of men (67%), with a mean BMI of 30 +/− 6.4 kg/m^2^. Regarding comorbidities: 68 (45%) had cardiovascular disease, 51 (34%) had respiratory disease, 16 (11%) had kidney chronic failure, and 52 (35%) had diabetes. Mean time to onset of COVID-19 symptoms was 7.5 days before hospitalization. Mean SOFA score on admission was 4 (+/−6) and mean SAPS II was 35 (+/−14).

**Table 1.** Patients characteristics and outcomes.

**Variables**	**Patients with sarcopenia n=75**	**Patients without sarcopenia n = 75**	**Total n = 150**	**p**
Age, years	69.6 (+/−10)	64.6 (+/−12.1)	67.1 (+/−11.4)	0.006
Male, n (%)	59 (78)	41 (41)	100 (66.7)	0.002

**Comorbidities**				
Respiratory, n (%)	25 (33)	26 (34.7)	51 (34)	0.863
Cardiac, n (%)	29 (38.7)	39 (52)	68 (45.3)	0.101
Renal, n (%)	6 (8)	10 (13)	16 (10.7)	0.290
Diabetes, n (%)	23 (30.7)	29 (38.7)	52 (34.7)	0.303
Obesity^**^, n (%)	19 (26)	50 (66.7)	69 (46.6)	<0.001
BMI, kg/m^2^	27.3 (+/−4.7)	33.1 (+/−6.6)	30.3 (+/−6.4)	<0.001

**CT scan variables**				
Lung damage, n (%):				
Mild (<25%)	21 (28)	21 (28)	42 (28)	0.050
Moderate (25–50%)	42 (56)	30 (40)	72 (48)	
Severe (>50%)	12 (16)	24 (32)	36 (24)	
Pulmonary embolism, n (%)	5 (6.8)	1 (1.3)	6 (4)	0.102
SMA, cm^2^	70.5 (+/− 16.6)	93.1 (+/−23.7)	83.3 (+/−23.8)	<0.001
SMI, cm^2^/m^2^	23.4 (+/− 4.5)	32.7 (+/−6.9)	28.8 (+/−7.5)	<0.001

**Scores**				
SAPS II	36.1 (+/−14.7)	33.7 (+/−12.1)	34.9 (+/−13.5)	0.267
SOFA	5.4 (+/−8.1)	3.6 (+/−2.9)	4.34 (+/−5.8)	0.089

**Biological parameters on ICU admission**				
Lymphocytes, G/L	0.84 (+/−0.83)	1.11 (+/− 1.22)	0.97 (+/−1.04)	0.112
Albumin, g/L	27.5 (+/−4.8)	29 (+/−5.3)	28.1 (+/−5.1)	0.266
CRP, mg/L	144.7 (+/− 116.2)	119.5 (+/− 89.3)	131.9 (+/−103.8)	0.155
D-Dimers, μg/L	1889.1 (+/−1632.6)	1291.42 (+/1311.5)	1598.5 (+/1508.5)	0.038
Fibrinogen, mmol/L	6.4 (+/−1.5)	6.9 (+/−1.6)	6.5 (+/−1.6)	0.624

**Outcomes**				
Mortality, n (%)	12 (16)	10 (13.3)	22 (14.7)	0.644
Pneumonia, n (%)	15 (20)	22 (29.3)	37 (24.7)	0.185
MV, n (%)	21 (28)	23 (37.3)	44 (29.3)	0.110
HFO duration, days	5.8 (+/−4.6)	4.0 (+/− 3)	4.9 (+/− 4)	0.005
MV duration, days	6.9 (+/− 9)	9.6 (+/− 12.1)	8.5 (+/−10.9)	0.233
ICU LOS, days	12.1 (+/− 9.7)	13.1 (+/− 12.2)	12.6 (+/− 11)	0.575
Hospital LOS, days	26.9 (+/− 22.8)	24.6 (+/− 20.6)	25.8 (+/− 21.7)	0.509

Data are presented as absolute values and percentages (n (%)) or mean (SD). BMI: Body Mass Index; HFO: High Flow Oxygen; LOS: Length Of Stay; MV: mechanical Ventilation; SMA: Skeletal Muscle Area; SMI: Skeletal Muscle Index.

** : combination of criterion 1 and a BMI > 30 kg/m^2^.

* : SMI<20.8 in r women and 28.8 in men or SMA <56.1 in women and 92.3 in men.

Regarding biological data on admission: mean lymphocyte level was 0.97 +/− 1.0 G/L, mean albumin level was 28.1 +/−5.1 g/L, mean CRP level was 131.9 +/−103.8 mg/L, mean D-Dimer level was 1598.5 +/−1508.5 μg/L, and mean fibrinogen level was 6.5 +/−1.6 mmol/L.

CT scans were performed 2.6 +/− 5 days after ICU admission, and the time between onset of symptoms and first CT scan was 9 +/− 6 days. Pulmonary embolism occurred in 6 patients (4%). Lung damages were defined as severe in 36 (24%) patients, moderate in 72 (48%) patients, and mild in 42 (28%) patients.

### Sarcopenia assessment (Table 1)

According to the gender-specific cutoffs at the T12 level, 75 (50%) patients had sarcopenia on ICU admission, despite a normal BMI for each. Mean SMI was 30.6 +/−7.4 cm^2^/m^2^ for men, and 25.1 +/−6.5 cm^2^/m^2^ for women. Mean BMI was 29.6 +/−5.9 kg/m^2^ for men, and 31.6 +/−7.2 kg/m^2^ for women.

### Outcomes in ICU (Table 1)

Overall, 22 (14.7%) patients died in ICU, 104 (71%) patients received HFO therapy for a mean duration of 5 +/− 4 days, and 44 (53%) were placed on MV for a mean duration of 8.5 +/−11 days. The mean LOS was 26 +/−22 days in hospital, and 12 +/−11 days in ICU.

### Impact of sarcopenia (Table 1)

There were more men in the sarcopenic group than in the non-sarcopenic group (59 (78%) *vs.* 41 (41%) p=0.002). Patients with sarcopenia were significantly older (70 *vs.* 65 years, p=0.006).

Regarding comorbidities in patients with sarcopenia: 29 (38.7%) had cardiac disease, 25 (33%) had respiratory disease, 6 (8%) had kidney chronic failure, and 23 (30.7%) had diabetes. There was no significant difference in the prevalence of these comorbidities between the groups with and without sarcopenia. There was more severe lung damage in the non-sarcopenic group (12 (16%) vs. 24 (32%); p=0.05).

Regarding outcomes, 12 (16%) patients with sarcopenia and 10 (13.3%) patients without sarcopenia died in ICU (p=0.644). Pneumonia occurred in 15 (20%) patients with sarcopenia and in 22 (29%) patients without, p=0.185. Mechanical ventilation was required in 21 (28%) patients with sarcopenia and in 23 (33%) patients without, p=0.11. HFO duration was significantly longer in patients with sarcopenia (6 *vs.* 4 days; p=0.005). MV duration was 6.9 (+/− 9) days in patients with sarcopenia and 9.7 (+/− 12.1) days in patients without, p=0.233. Mean ICU LOS was 12,1 (+/− 9.7) days in patients with sarcopenia and 13.1 (+/− 12.2) days in patients without, p=0.575. Mean hospital LOS was 26.9 (+/− 22.8) days in patients with sarcopenia and 24.6 (+/− 20.6) days in patients without sarcopenia, p = 0.509.

### Impact of sarcopenia in high flow oxygen therapy patients' subgroup (Table 2)

Among 104 patients who received high flow oxygen therapy, there were 54 patients with sarcopenia and 50 patients without. There were 42 (78%) men with sarcopenia and 26 (52%) without, p=0.006. The mean BMI was 27 +/− 4.9 kg/m^2^ in patients with sarcopenia, and 32.7 +/−6.7 kg/m^2^ in patients without, and the mean age 69 +/− 11 years versus 64 +/− 13 years, p=0.032.

**Table 2.** Effect of sarcopenia on High flow oxygen

**Variables**	**Patients with sarcopenian =54**	**Patients without sarcopenia n = 50**	**Total n = 104**	**p**
Age, years	69.1 (+/−1)	63.9 (+/−13.4)	66.6(+/−12.4)	0.032
Male, n (%)	42 (77.8)	26 (52)	68 (65.4)	0.006

**Comorbidities**				
Respiratory, n (%)	17 (31.5)	21 (42)	38 (36.5)	0.266
Cardiac, n (%)	19 (35.2)	25 (50)	44 (42.3)	0.127
Renal, n (%)	3 (5.6)	4 (8)	7 (6.7)	0.619
Diabetes, n (%)	18 (33.3)	19 (38)	37 (35.6)	0.619
BMI, kg/m^2^	27 (+/− 4.9)	32.71 (+/−6.7)	29.8 (+/−6.5)	<0.001

**CT-Scan variables**				
Lung damage, n (%):				
Mild (<25%)	17 (31.5)	15 (30)	32 (30.8)	0.795
Moderate (25–50%)	29 (53.7)	24 (48)	53 (51)	
Severe (50–75%)	7 (13)	9 (18)	16 (15.4)	
Very severe (>75%)	1 (1.9)	2 (4)	3 (2.9)	
Pulmonary embolism, n (%)	4 (7.4)	1 (2)	5 (4.8)	0.198

**Scores**				
SAPS II	36.2 (+/−12.8)	28.8 (+/−9.2)	32.6 (+/−11.7)	0.001
SOFA	3.4 (+/−2.1)	2.6 (+/−1.7)	2.9 (+/−1.9)	0.084

**Biological parameters on ICU admission**				
Lymphocytes, G/L	0.9 (+/−0.9)	1.1 (+/− 1.4)	1.0 (+/−1.2)	0.298
Albumin, g/L	29.2 (+/−4.9)	29.5 (+/−3.3)	29.3(+/−4.4)	0.859

**Outcomes**				
Mortality, n (%)	6 (11.1)	4 (8)	10 (9.6)	0,421
Pneumonia, n (%)	1 (1.9)	1 (2)	2 (1.9)	0.733
HFO duration, days	6.8 (+/−4.4)	5 (+/− 2.9)	5.9 (+/−3.8)	0.005
ICU LOS, days	8.2 (+/− 5)	6.3 (+/− 3.1)	7.3 (+/− 4.3)	0.019
Hospital LOS, days	21.8 (+/− 17.6)	17.1 (+/− 14.2)	19.3 (+/−16.1)	0.143

Data are presented as absolute values and percentages (n (%)) or mean (SD). BMI: Body Mass Index; HFO: High Flow Oxygen; LOS: Length Of Stay.

**Table 3.** Effect of mechanical ventilation at admission

**Variables**	**Mechanical ventilation on admission n =13**	**No mechanical ventilation on admission n = 137**	**Total n = 150**	**p**
Age, years	67.1 (+/−11.4)	67.13 (+/−11.4)	67.13(+/−11.3)	0.998
Male, n (%)	6 (46.2)	94 (68.6)	100 (66.7)	0.101

**Comorbidities**				
Respiratory, n (%)	3 (23.1)	48 (35)	51 (34)	0.294
Cardiac, n (%)	6 (46.2)	62 (45.3)	68 (45.3)	0.950
Renal, n (%)	1 (7.7)	15 (10.9)	16 (10.7)	0.584
Diabetes, n (%)	5 (30.8)	48 (35)	52 (34.7)	0.509
Sarcopenia, n (%)	4 (30.8)	71 (51.8)	75 (50)	0.123
BMI, kg/m^2^	33.1(+/− 8)	30.0 (+/−6.2)	30.3 (+/−6.4)	0.101

**CT-Scan variables**				
Lung damage, n(%):				
Mild (<25%)	3 (23.1)	39 (28.5)	42 (28)	0.062
Moderate (25–50%)	3 (23.1)	69 (50.4)	72 (48)	
Severe (50–75%)	6 (46.2)	24 (17.5)	30 (20)	
Very severe (>75%)	1 (7.7)	5 (3.6)	6 (4)	
Pulmonary embolism, n (%)	0 (0)	6 (4.4)	6 (4)	0.573

**Scores**				
SAPS II	46.8 (+/−18.7)	33.8 (+/−12.1)	34.9 (+/−13.5)	0.001
SOFA	6.0 (+/−4.2)	4.2 (+/−6)	4.3 (+/−5.8)	0.276

**Biological parameters at admission**				
Lymphocytes, G/L	0.96 (+/−0.8)	0.97 (+/− 1.1)	0.97 (+/−1.1)	0.968
Albumin, g/L	28.8 (+/−8.1)	28.0 (+/−4.5)	28.1 (+/−5.1)	0.670

**Outcomes**				
Mortality, n (%)	3 (23.1)	19 (13.9)	22 (14.7)	0.292
Pneumonia, n (%)	11 (84.6)	26 (19)	37 (24.7)	<0.001
MV duration, days	19.0 (+/− 9.6)	6.8 (+/− 10.2)	8.5 (+/−10.9)	<0.001
ICU LOS, days	23.7 (+/− 13.28)	11.5 (+/− 10.2)	12.6 (+/− 11.0)	0.001
Hospital LOS, days	34.0 (+/− 20.4)	25.0 (+/− 21.7)	25.8 (+/− 21.7)	0.154

Data are presented as absolute values and percentages (n (%)) or mean (SD). BMI: Body Mass Index; LOS: Length Of Stay; MV: mechanical Ventilation

**Table 4.** Effect of sarcopenia in obese patients

**Variables**	**Obese patients with sarcopenia n =19**	**Obese patients without sarcopenia n = 50**	**Total n = 69**	**p**
Age, years	69.8 (+/−10.5)	63.17 (+/−12.8)	65 (+/−12.5)	0.048
Male, n (%)	14 (73.7)	28 (56)	32 (60.9)	0.179

**Comorbidities**				
Respiratory, n (%)	9 (47.4)	22 (44.0)	31 (44.9)	0.802
Cardiac, n (%)	8 (42.1)	27 (54)	35 (50.7)	0.377
Renal, n (%)	3 (15.8)	10 (20)	13 (18.8)	0.689
Diabetes, n (%)	8 (42.1)	24 (48)	32 (46.4)	0.661
BMI, kg/m^2^	33.2 (+/− 4.5)	36.3 (+/−5.7)	35.5 (+/−5.6)	0.036

**CT scan variables**				
SMA, cm^2^	66.4 (+/− 12.1)	99.5 (+/−25)	90.9 (+/−27.3)	<0.001
SMI, cm^2^/m^2^	23.0 (+/− 7.6)	34.9 (+/−7.4)	31.79 (+/−8.6)	<0.001

**Scores**				
SAPS II	35.8 (+/−17.6)	34.1 (+/−11.9)	34.6 (+/−13.6)	0.652
SOFA	7.2 (+/−11.6)	3.8 (+/−2.8)	4.5 (+/−5.8)	0.069

**Biological parameters at admission**				
Lymphocytes, G/L	1.00 (+/−1.4)	1.01 (+/− 0.6)	1.01 (+/−0.9)	0.971
Albumin, g/L	28.8 (+/−6)	28.7 (+/−6)	28.8 (+/−6)	0.94

**Outcomes**				
Mortality, n (%)	4 (21.1)	5 (10)	9 (13)	0.223
Pneumonia, n (%)	6 (31.6)	16 (32)	22 (31.9)	0.973
MV, n (%)	7 (36.8)	18 (36.0)	25 (36.2)	0.984
HFO duration, days	4.6 (+/−3.3)	3.6 (+/− 2.7)	3.9 (+/−2.9)	0.207
MV duration, days	8.0 (+/− 8.1)	11.8 (+/− 13.5)	10.8 (+/−12.4)	0.367
ICU LOS, days	12.5 (+/− 8.7)	14.7 (+/− 14.2)	14.1 (+/− 12.9)	0.518
Hospital LOS, days	263.8 (+/− 22.4)	25.5 (+/− 21.3)	25.1 (+/− 21.5)	0.768

Data are presented as absolute values and percentages (n (%)) or mean (SD). BMI: Body Mass Index; HFO: High Flow Oxygen; LOS: Length Of Stay; MV: mechanical Ventilation; SMA: Skeletal Muscle Area; SMI: Skeletal Muscle Index.

Regarding comorbidities in patients with sarcopenia: 19 (35.2%) had cardiac disease, 17 (31.5%) had respiratory disease, 3 (6%) had kidney chronic failure, and 18 (33%) had diabetes. There was no significant difference in the prevalence of these comorbidities between the HFO subgroups with and without sarcopenia. Patients with sarcopenia had higher SAPSII scores (36 *vs.* 29 p=0.001), with no significant difference in SOFA scores (3.4 vs. 2.6, p=0.084).

There was no significant difference between the two populations regarding biological data, such as lymphocyte and albumin levels (p=0.298 and p=0.859, respectively). Regarding lung damage, there was no association between sarcopenia and the severity. Pulmonary embolism was present in 4 (7.4%) patients with sarcopenia and 1 (2%) without, p=0.198. There was only one pneumonia event in each population.

HFO duration was longer in patients with sarcopenia (7 +/− 4 days vs. 5 +/−3 days, p=0. 005), as was ICU LOS (8 +/− 5 days *vs.* 6 +/−3 days p=0.019). Hospital LOS was not statistically different (22 +/−18 days *vs.* 17 days +/− 14 days p=0.143). In this population, 6 (11%) patients with sarcopenia and 4 (8%) patients without sarcopenia died in ICU (p= 0.589). No death occurred after ICU stay.

### Impact of sarcopenia in ventilated patients' subgroup (table 3)

Thirteen (9%) patients were placed on MV on ICU admission. Among them, 6 (46.2%) were men. Compared with the overall population, ventilated patients were not significantly older (67 years, p=0.998). In this subgroup, 6 (46.2%) had heart disease, 3 (23.1%) had respiratory disease, 1 (7.7%) had kidney chronic disease and 5 (30.8%) had diabetes. There was no significant difference in the prevalence of these comorbidities between the MV subgroup and the overall population, nor in the prevalence of sarcopenia (4 (30.8%) vs. 71 (51.8%); p=0.123).

Regarding lung damage, there were no significant differences in the severity states between ventilated and non-ventilated patients (p=0.062), nor in lymphocyte and albumin levels (p=0.968 and p=0.670, respectively).

MV duration was longer for patients placed on MV on admission (19 +/−9.6 day *vs.* 7 +/− 10.2 days, p<0.001), as was the ICU LOS (24 +/− 13 days *vs.* 12 +/−10 days p<0.001). Hospital LOS was not significantly different between the two groups (34 +/− 20 days *vs.* 25 +/− 22 days p = 0.154). Mortality was not statistically different between the two groups (p=0.292).

### Impact of sarcopenia in high obese patients' subgroup (table 4)

Among 69 obese patients included, 19 (25%) patients had sarcopenia. The mean age was significantly higher in obese patients with sarcopenia (70 +/− 11 years vs. 63 +/− 13 years; p=0.048), and their mean BMI was lower (33 +/− 5 kg/m^2^ *vs.* 36 +/− 6 kg/m^2^ p=0.036).

There were no significant differences in terms of comorbidities, severity of lung damage, lymphocyte or albumin levels between obese patients with sarcopenia and those without. Also, there were no significant differences in mortality, pneumonia incidence or MV use (p=0.223, p=0.973 and p=0.948, respectively). Similarly, for HFO duration, MV duration, ICU and hospital LOS (p=0.207, p=0.367, p=0.518 and p=0.768, respectively).

Of the 22 deaths in the overall population, 9 (41%) were obese patients.

## Discussion

In this retrospective study, we evaluated the association between pre-existing sarcopenia and outcomes in critically ill patients admitted to the ICU for ARF secondary to COVID-19. In our population, we found a major prevalence (50%) of pre-existing sarcopenia, and we observed a significant impact of sarcopenia on the HFO duration, but not on other outcomes (MV requirement and duration, ICU mortality and hospital LOS). Also, no differences in outcomes were observed between sarcopenic and non-sarcopenic obese patients.

The high prevalence we found seems to be consistent with the data in the literature. Indeed, studies considering all COVID-19 patients admitted to ICU reported a variable prevalence of sarcopenia, ranging from 24% to 40%, while those considering only mechanically ventilated COVID-19 patients reported a higher prevalence of around 65% [20,21,22].

In our study, sarcopenia was not associated with other comorbidities such as diabetes or hypertension, unlike in other studies [20,21]. However, it is worth noting that some of these studies do not systematically find links between sarcopenia and these two comorbidities, possibly illustrating the population heterogeneity among ICU admission during the pandemic worldwide. On the other hand, our data are consistent with regard to obesity, which is more frequent in the nonsarcopenic group [21].

Regarding outcomes, we observed a higher HFO duration in the sarcopenic group, but the presence of pre-existing sarcopenia did not appear to influence the need for MV, LOS or mortality. Beyond COVID-19 disease, the impact of sarcopenia on the outcome of intensive care patients, whatever their pathology, seems clear. A study conducted in 362 patients between 2012 and 2017 for medical and surgical conditions reported an increase in mortality related to sarcopenia [4]. A retrospective study of 905 patients treated for septic shock showed an increase in short-, medium- and long-term mortality in 407 patients with sarcopenia [23]. A follow-up of 72 post-surgical ICU patients demonstrated an excess risk of one year mortality [24], with delayed ventilatory withdrawal and increased mortality in the presence of sarcopenia at the end of ICU stay [25]. However, when focusing on ICU patients admitted with COVID-19 ARF, the impact of sarcopenia appears less obvious. A recent meta-analysis including almost 500,000 patients reported an increased risk of mortality and severe lung damage for sarcopenic patients [26]. In addition, Damanti *et al.* showed easier ventilatory withdrawal and shorter hospital LOS in patients without sarcopenia [22]. In contrast, and surprisingly as in our study where the MV duration did not differ between the two groups, other studies did not report deleterious effects due to sarcopenia. Molwitz *et al.* found no correlation between sarcopenia and duration of ventilation, ICU LOS, or mortality [27]. Another study of 207 COVID-19 patients admitted to the ICU, including 49 (24%) patients with sarcopenia, found no difference between patients with and without sarcopenia concerning the requirement for MV, MV duration or mortality [21]. These authors suggested a possible underestimation of muscle mass measured at T12, compared to the classic L3 level, because of the presence of a positive fluid balance due to edema. The absence of an association between sarcopenia and mortality was also reported in a single-center Italian study of 25 patients [28].

As regards mechanical ventilation, its duration did not appear to be higher in sarcopenic patients. This result, although contradictory to the physiopahological logic of ventilatory weaning, and most of the data in the literature (cited above), was also observed in a recent study of 472 adult polytrauma patients [29]. Inconsistencies in this result could come from the diagnostic method used to define sarcopenia. Indeed, as shown in a recent meta-analysis, the CT diagnosis can be established on the analysis of total skeletal muscle mass at levels ranging from the T4 to L4 vertebrae, and sometimes even on the total psoas muscle area [30].

Interestingly, some studies showed a “positive” impact of sarcopenia on patient outcome: a meta-analysis on patients with ovarian cancers reported a negative relationship between muscle mass and mortality [31]. The impact of sarcopenia appears to be paradoxical, depending on the patient's phenotype and underlying disease. Numerous studies have shown the negative impact of high perivisceral fat mass on COVID-19 severity [32,33,34,35], an excess mortality rate in obese patients with COVID-19 pneumonia [36,37], suggesting different mechanisms such as a decrease in immunity, pro-inflammatory process, and a high level of angiotensin converting enzyme receptor in adipocytes [38,39]. Patients with COVID-19 pneumonia had a 2-fold higher level of angiotensin-converting enzyme in the diaphragm compared to non-COVID-19 patients. However, angiotensin-converting enzyme has a pro-fibrosing effect on the diaphragm, and is secreted in proportion to fat mass [40], which may explain longer MV duration in obese patients. In our population, while one might think of a cumulative negative effect of sarcopenia with obesity on patient outcome [41], such is not the case, since we found no difference between outcome of obese patients with sarcopenia and those without. Similar results were found in the study by Molfino *et al*. [42]. Sarcopenia, like cancer, seems to be a condition where the obesity paradox would come into the equation regarding COVID-19 population [43].

Our study has several limitations. First, because of its retrospective design, the risk of selection bias was inevitable. Second, survival analysis did not reach statistical significance due to a lack of power with an insufficient number of patients analyzed and some confounding factors (severity of underlying COVID, severity at admission to ICU, lack of data on nutritional support in ICU). In addition, given the modest mortality rate in our study, compared to that worldwide, we can hypothesize that most frail or severely malnourished patients were not admitted to intensive care due to the triage required for the massive influx of patients during the epidemic [44]. This could explain the lack of significance of morbidity and mortality parameters. Third, it is possible that muscle mass at T12 has been underestimated compared to the classic L3 level. Although many studies use this same reference, only the mass at the L3 level was compared to the reference test for the diagnosis of sarcopenia (i.e. dual-energy X-ray absorptiometry). Fourth, the absence of muscle function testing did not allow sarcopenia to be diagnosed with certainty, but only detected by a decrease in lean mass. Finally, it should be noted that we assessed the presence of sarcopenia only at the time of ICU admission and not during the stay, which does not allow us to know whether severe COVID-19 is a factor favoring sarcopenia.

## Conclusion

In critically ill patients with COVID-19 acute respiratory failure, we observed a high prevalence of pre-existing sarcopenia with a higher HFO duration. However, the impact of sarcopenia on other outcomes remains unclear. Future studies are required to compare outcomes of sarcopenic patients using varied diagnostic criteria. These studies should also enable a more comprehensive assessment of body composition and long-term functional status in this specific population.

## Abbreviations

ARF: Acute Respiratory Failure
BMI: Body Mass Index
COVID-19: Coronavirus disease 2019
CRP: C-reactive protein
CSA: Cross-Sectional Area
CT: Computed Tomography
HFO: High Flow Oxygen
HU: Hounsfield Unit
ICU: Intensive Care Unit
LOS: Length Of Stay
MV: Mechanical Ventilation
RT-PCR: Reverse transcription polymerase chain reaction
SAPS II: Simplified Acute Physiology Score II
SMA: Skeletal Muscle Area
SMI: Skeletal Muscle Index
SOFA score: Sequential Organ Failure Assessment Score
